# Association between adherence to calcium-channel blocker and statin medications and likelihood of cardiovascular events among US managed care enrollees

**DOI:** 10.1186/1471-2261-10-29

**Published:** 2010-06-17

**Authors:** Richard H Chapman, Jason Yeaw, Craig S Roberts

**Affiliations:** 1 US Health Economics & Outcomes Research, IMS Health, Falls Church, VA, USA; 2 Global Outcomes Research, Pfizer Inc, New York, NY, USA

## Abstract

**Background:**

Prior studies have found that patients taking single-pill amlodipine/atorvastatin (SPAA) have greater likelihood of adherence at 6 months than those taking 2-pill calcium-channel blocker and statin combinations (CCB/statin). This study examines whether this adherence benefit results in fewer cardiovascular (CV) events.

**Methods:**

A retrospective cohort study was conducted using administrative claims data from the IMS LifeLink: US Health Plan Claims database, identifying adults already taking CCB or statin (but not both) who had an index event of either initiating treatment with SPAA or adding CCB to statin (or vice versa) between April 1, 2004 to August 31, 2005. Inclusion criteria included age 18+ years, continuously enrolled for minimum of 6 months prior and 18 months following treatment initiation, >1 diagnosis of hypertension, and no prescription claims for SPAA or added CCB or statin for 6 months prior. Exclusion criteria included >1 claim with missing or invalid days supplied, age 65+ years and not enrolled in Medicare Advantage, or history of prior CV events, cancer diagnosis, or chronic renal failure. The primary outcome measure was the rate of CV events (myocardial infarction, heart failure, angina, other ischemic heart disease, stroke, peripheral vascular disease, or revascularization procedure) from 6 to 18 months following index date, analyzed at three levels: 1) all adherent vs. non-adherent patients, 2) SPAA vs. dual-pill patients (regardless of adherence level), and 3) adherent SPAA, adherent dual-pill, and non-adherent SPAA patients vs. non-adherent dual-pill patients.

**Results:**

Of 1,537 SPAA patients, 56.5% were adherent at 6 months, compared with 21.4% of the 17,910 CCB/statin patients (p < 0.001). Logistic regression found SPAA patients more likely to be adherent (OR = 4.7, p < 0.001) than CCB/statin patients. In Cox proportional hazards models, being adherent to either regimen was associated with significantly lower risk of CV event (HR = 0.77, p = 0.003). A similar effect was seen for SPAA vs. CCB/statin patients (HR = 0.68, p = 0.02). In a combined model, the risk of CV events was significantly lower for adherent CCB/statin patients (HR = 0.79, p = 0.01) and adherent SPAA patients (HR = 0.61, p = 0.03) compared to non-adherent CCB/statin patients.

**Conclusions:**

Patients receiving SPAA rather than a 2-pill CCB/statin regimen are more likely to be adherent. In turn, adherence to CCB and statin medications is associated with lower risk of CV events in primary prevention patients.

## Background

CVD is the number one cause of death globally and will remain so, taking an estimated 20 million lives annually by 2015 [1]. Two of the most prevalent and modifiable risk factors for CVD -- hypertension and dyslipidemia -- commonly coexist. The risk of CVD is greater in people with both of these risk factors than it is in those with either condition alone [2,3]. Effective treatment of these two CVD risk factors is widely available and has been proven to reduce CV events. The benefits of antihypertensive medications and 3-hydroxy-3-methylglutaryl-coenzyme A reductase inhibitors (statins) for reducing CHD and stroke risk in patients at a high risk of CHD have been demonstrated in several well-known clinical trials [4,5]. Also, meta-analyses have shown the consistent effects from antihypertensive [6] and statin [7-9] medications in reducing CV events. Despite these effective treatments for hypertension and dyslipidemia, and the associated reduction in CV events, control of these conditions often remains suboptimal, partly due to poor patient adherence [10].

Recent analyses report that fixed dose combination (FDC) therapy for hypertension and dyslipidemia is associated with a greater likelihood of adherence than the historic approach of prescribing medication for each risk factor separately [10,11]. For example, patients taking single-pill amlodipine/atorvastatin (SPAA) have a greater likelihood of adherence at 6 months than those taking 2-pill calcium-channel blocker and statin combinations (CCB/statin) [10]. Other studies show that when two-pill CCB/statin regimens are initiated close together in time, adherence is greater than when therapy is initiated sequentially, [12-14] and that, in general, adherence is better with single-pill regimens vs. 2-pill regimens [15,16]. The reasons for better adherence with FDC therapy for hypertension and dyslipidemia may include reduced pill burden [17] and reduced patient-borne medication costs [18,19].

Efforts to improve patient adherence to CVD medication therapy are important, as retrospective analyses have shown that adherence to statins and to antihypertensive medications have been associated with reduced rates of CV events [20-23]. In a recent review of the literature, poor compliance with lipid-lowering treatment has been shown to be associated with poorer clinical outcomes and increased cardiovascular morbidity and mortality [20]. Bouchard et al. [21], using a nested case-control design, found that adherence to statins that exceeded 90% was associated with a significant reduction in nonfatal CAD events after one year of treatment. Another nested case-control analysis, by Perreault et al. [22], found that high adherence levels to antihypertensive therapy were associated with relative risk reduction in CAD events compared to low levels of adherence. Mazzaglia et al. [23] reported a similar finding among newly diagnosed hypertensive patients in a retrospective cohort analysis. To build upon the growing body of evidence supporting the impact of adherence on reduction in CV events, this study examines whether the adherence benefit previously demonstrated with SPAA results in fewer CV events than for patients on 2-pill regimens.

## Methods

We conducted a retrospective cohort study using administrative claims that include medical and pharmacy data from the IMS LifeLink: US Health Plan Claims database for October 1, 2003 through August 31, 2006. The database is comprised of fully adjudicated medical & pharmaceutical claims for over 65 million unique patients from over 90 health plans across the US (with approximately 16 million covered lives per year). It includes both inpatient and outpatient diagnoses and procedures as well as prescription records, and is generally representative of the national, commercially-insured population in terms of age, gender, and type of health plan. The data is longitudinal, with average member enrollment duration of nearly two years. Only health plans that submit data for all members are included in the database, ensuring complete data capture & representative samples. The data are subjected to a series of quality checks to ensure standardized format & minimal error rates.

### Study population

We identified adults taking CCB or statin (but not both) who then initiated treatment with SPAA or added CCB to statin (or vice versa) from April 1, 2004 to August 31, 2005. Inclusion criteria included age ≥18 years, at least one prescription for SPAA or CCB + statin (with first prescription for SPAA or the added CCB or statin in the study period considered the index date), continuously enrolled for minimum of 6 months prior to and 18 months following index date, >1 diagnosis of hypertension prior to or on the index date, and no claims for the index prescription(s) for 6 months prior to index date. Exclusion criteria included at least one prescription claim with missing or invalid days supplied, age 65 years or greater and not enrolled in Medicare Advantage, or history of prior CV events, cancer diagnosis, or chronic renal failure. Patients were considered secondary prevention patients if they had evidence during the pre-index period of any of the specified CV-related events or procedures, and were excluded from analysis. Otherwise, patients' treatment was considered to be for primary prevention.

**Figure 1:**
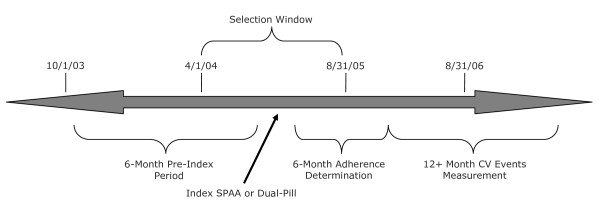
Illustration of the study time periods

This study included 3 time periods, as illustrated in Figure 1:

1. Pre-index: 6-month period in which patients were taking either statin *or *CCB

2. Adherence measurement period: 6-month period following initiation of SPAA or dual therapy (index) where adherence is assessed

3. Follow-up period: ≥12 months in which CV events are tabulated

### Adherence

The proportion of days covered (PDC) for each of the two drug cohorts was calculated for the 6-month adherence measurement period. Adherence was capped at 100%, and calculated as the total days supplied of index drug divided by the number of days in the follow-up period (a denominator of 180 days). Claims extending beyond day 179 were pro-rated to include only the portion of days' supply captured within the observation period. Additionally, if a patient refilled a prescription early, any days with overlapping prescriptions were counted only once. Advantages of using PDC as an adherence measure are that it simultaneously reflects both compliance and persistence, [24,25] and is a commonly used measure in adherence studies [12,26-30], allowing useful comparisons of adherence levels across studies.

For this analysis, patients were considered "adherent" if PDC by SPAA or CCB *and *statin was ≥80%, and non-adherent if PDC <80% for the 6-month period. Unadjusted adherence rates of patients in the two treatment groups are reported for the 6-month follow-up period. Multivariable logistic regression models with adherence status (</≥80% PDC) as the dependent variable were also run.

### **Study Outcomes**

The primary outcome of interest was the rate of CV events, as well as its relationship to 6-month adherence levels. CV events were defined as hospitalization for myocardial infarction (MI), heart failure, angina, other ischemic heart disease, stroke, peripheral vascular disease, or CV revascularization procedure. CV event definition included events with either a primary discharge diagnosis of interest or a procedure of interest using only inpatient claims; outpatient claims were not considered. To allow sufficient time for events of interest to occur, this analysis was restricted to patients with at least 18 months of continuous enrollment following their medication-based index date.

Rates of CV events were analyzed at three levels:

1) all adherent patients vs. all non-adherent patients;

2) SPAA patients vs. dual-pill patients (regardless of adherence level);

3) adherent SPAA patients, adherent dual-pill patients, and non-adherent SPAA patients vs. non-adherent dual-pill patients.

All CV events were reviewed starting at 180 days post-index (to allow a 6-month period for the establishment of adherence) and ending with patient disenrollment or the end of the study period. Any CV events that may have occurred in the first 180 days post-index were ignored for the purpose of this analysis. CV events were defined as the presence of claims with an ICD-9 code for a relevant diagnosis or a CPT-4 code for a procedure of interest, which were: myocardial infarction (MI, ICD-9 410.xx, 412), other ischemic heart disease including unstable angina (411.xx, 414.xx, 427.xx, V45.81, V45.82), stroke/TIA (433.xx, 434.xx, 435.x, 436, 437.0, 437.1, 438), peripheral vascular disease (440, 440.1, 443.xx), angina with hospitalization (413.xx), coronary artery bypass graft (CABG, CPT-4 code 33503 - 33545), carotid endarterectomy (35301, 35390, 35901), coronary stenting (92980, 92981), percutaneous transluminal coronary angioplasty (PTCA)/thrombectomy/atherectomy (92973, 92982, 92984, 92995, 92996), or percutaneous transluminal pulmonary artery balloon angioplasty (92997, 92998). Only the first CV event in the observation period per patient was included in the analysis. The total number of events overall and in each of the treatment groups are reported. Additionally, the rate of CV events was calculated as the total number of events divided by the total amount of patient-time contributed to the analysis for each treatment group. Patient-time was allowed to vary, with a minimum value of 360 days per patient. The crude rates of events (total events divided by total person-days) are reported overall and for each cohort.

In addition to the crude rates described above, the adjusted CV event rates for all patients and by treatment group were determined using Cox proportional hazards models, with covariates to account for potentially confounding factors. The dependent variable was days to CV event. Independent variables included all relevant demographic and clinical characteristics.

### Statistical analyses

Time to CV event was plotted using the Kaplan-Meier estimator. To adjust for differences in patient characteristics for each treatment group, the time to CV event was also modeled using a Cox proportional hazards model, with days from index date to CV event as the dependent variable. Independent variables included therapy type (SPAA vs. combination), adherence status, gender, age group, geographic region, health plan type, insurance type, related pre-index comorbidities, and number of pre-index antihypertensive classes being taken.

## Results

### Patient characteristics

**Figure 2:**
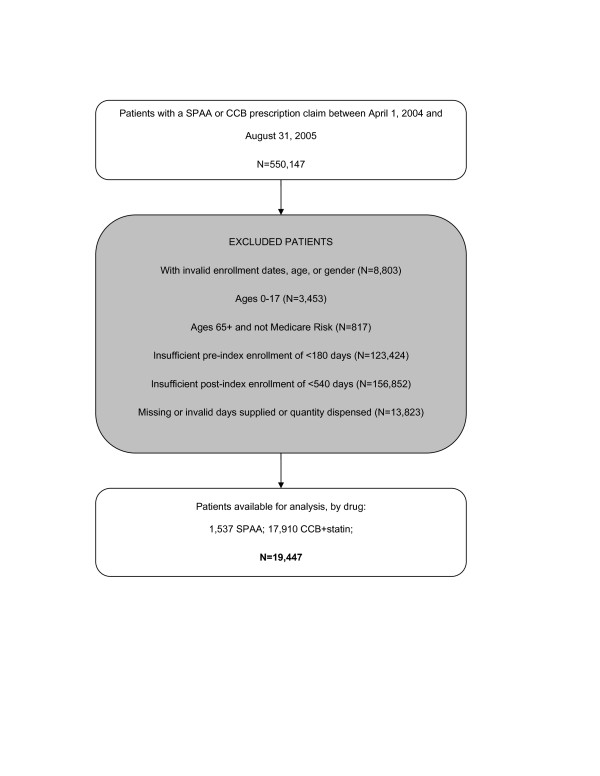
Study cohort identification procedure, with reasons for inclusion/exclusion through the selection process

As shown in Figure 2, after applying our inclusion and exclusion criteria, 19,447 patients were available for analysis; Table 1 details the demographic and clinical characteristics of these patients. The mean (SD) age was 53.5 (7.6) years for SPAA patients and 54.8 (8.5) years for the CCB+statin patients (p < 0.001). SPAA patients were more likely to be male than CCB+statin patients (58.6% vs. 52.1%, respectively; p < 0.001). SPAA patients were less likely to have diabetes but more likely to have a dyslipidemia diagnosis than were CCB+statin patients, and on average were taking fewer other medications pre-index (Table 1).

**Table 1:** Demographic and clinical characteristics of SPAA and CCB+statin primary prevention patients

	SPAA		CCB+Statin		
Characteristic	(N = 1,537)		(N = 17,910)		p-value
**Age: (Years)**					
Mean	53.5		54.8		<.001
SD	7.6		8.5		
Median	55		55		
					
**Age group: (n,%)**					<.001
<50 years	412	26.8%	4,119	23.0%	
50-59 years	816	53.1%	9,636	53.8%	
60+ years	309	20.1%	4,155	23.2%	
					
**Gender: (n,%)**					
Male	900	58.6%	9,327	52.1%	<.001
					
**Geographic Region: (n,%)**					<.001
Northeast	525	34.2%	4,705	26.3%	
Midwest	389	25.3%	7,314	40.8%	
South	595	38.7%	5,296	29.6%	
West	28	1.8%	595	3.3%	
					
**Plan Type: (n,%)**					<.001
Health Maintenance Organization (HMO)	311	20.2%	6,429	35.9%	
Indemnity Plan	92	6.0%	885	4.9%	
Point of Service (POS)	217	14.1%	2,313	12.9%	
Preferred Provider Organization (PPO)	884	57.5%	7,805	43.6%	
Unknown	33	2.1%	478	2.7%	
					
**Payer Type: (n,%)**					<.001
Commercial Plan	1,462	95.1%	16,121	90.0%	
Medicaid	1	0.1%	158	0.9%	
Medicare Risk	26	1.7%	1,086	6.1%	
Self-Insured	38	2.5%	464	2.6%	
Other/Unknown	10	0.7%	81	0.5%	
					
**Charlson Comorbidity Burden**:					
Mean	0.4		0.5		<.001
SD	0.7		0.8		
					
**Comorbid Conditions of Interest: (n,%)**					
Diabetes Mellitus	363	23.6%	5,242	29.3%	<.001
Dyslipidemia	1,188	77.3%	12,217	68.2%	<.001
Obesity	76	4.9%	1,063	5.9%	0.112
Peripheral Vascular Disease	35	2.3%	476	2.7%	0.371
Chronic Obstructive Pulmonary Disease	39	2.5%	638	3.6%	0.035
					
**Number of Pre-Index Unique Prescriptions**:					<.001
1-2	252	16.4%	1,847	10.3%	
3-4	392	25.5%	3,646	20.4%	
5-6	310	20.2%	3,682	20.6%	
7+	583	37.9%	8,735	48.8%	
					
**Number of Pre-Index Other Antihypertensive Classes**:					<.001
0	675	43.9%	6,771	37.8%	
1	718	46.7%	9,237	51.6%	
2	127	8.3%	1,693	9.5%	
3+	17	1.1%	209	1.2%	

### Adherence

Of the 1537 SPAA patients, 56.5% were adherent (PDC ≥80%) at 6 months, compared with 21.4% of the 17,910 CCB+statin patients (Table 2). Although adherence continued to decline over time in both groups, the percentage of patients adherent remained significantly higher in the SPAA group than in the CCB+statin group at 18 months (42.3% vs. 18.7%, respectively; p < 0.001). After adjusting for patients' clinical and demographic characteristics (as listed above), SPAA patients were significantly more likely to be adherent (OR = 4.7, p < 0.001, Table 3) than CCB/statin patients, as were patients with dyslipidemia (OR = 1.2).

**Table 2:** Adherence measures for SPAA and CCB+statin primary prevention patients

	SPAA		CCB+Statin		
	(N = 1,537)		(N = 17,910)		p-value
**6 Months**					
Number (%) of patients with PDC ≥80%	868	56.5%	3,825	21.4%	<.001
Mean PDC (SD)	0.73	(0.26)	0.49	(0.31)	
Median PDC	0.83		0.50		<.001
					
**12 Months**					
Number (%) of patients with PDC ≥ 80%	712	46.3%	3,529	19.7%	<.001
Mean PDC (SD)	0.66	(0.30)	0.46	(0.31)	
Median PDC	0.75		0.46		<.001
					
**18 Months**					
Number (%) of patients with PDC ≥ 80%	650	42.3%	3,342	18.7%	<.001
Mean PDC (SD)	0.62	(0.31)	0.43	(0.32)	
Median PDC	0.72		0.42		<.001

**Table 3:** Logistic regression model of medication adherence (PDC ≥80%) at 6 months following initiation of SPAA or CCB+statin

		95% Confidence Limits			
	Odds Ratio	Lower	Upper	Chi-Square	p-value
Drug Group: SPAA vs. CCB+Statin	4.70	4.22	5.23	793.06	<.001
Gender: Female vs. Male	1.21	1.13	1.30	30.59	<.001
Pre-index Comorbidity of:					
Diabetes Mellitus	1.00	0.92	1.07	0.02	0.891
Dyslipidemia	1.22	1.14	1.32	27.80	<.001
Obesity	0.84	0.72	0.98	5.15	0.023
Peripheral Vascular Disease	0.75	0.59	0.94	6.26	0.012
Chronic Obstructive Pulmonary Disease	0.82	0.67	0.99	4.17	0.041
Number of Pre-Index Other Antihypertensive Classes	1.12	1.06	1.18	18.93	<.001

### CV event rates

**Figure 3:**
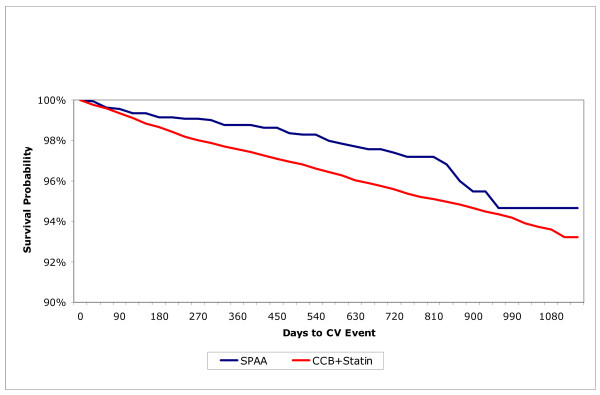
Kaplan-Meier analysis of days to CV event in SPAA and CCB/statin primary prevention patients with no history of cancer or chronic renal failure

The crude (unadjusted) CV event rate for each patient stratification is shown in Table 4. Non-adherent patients and CCB/statin patients experienced higher CV event rates than adherent and SPAA patients, respectively (Table 4). A similar pattern was observed when time to CV event was examined in Kaplan-Meier analyses (Figure 3).

**Table 4:** CV events from 6 months following initiation of SPAA or CCB+statin in primary prevention patients

	Overall	Adherent	Non-Adherent	SPAA	CCB+Statin
N=	(19,447)	(4,693)	(14,754)	(1,537)	(17,910)
**12-month Event Rate**					
Total Events (N)	452	88	364	19	433
Total Person-Years	19,447	4,693	14,754	1,537	17,910
Incidence Rate per 100 person-years	2.32	1.88	2.47	1.24	2.42
					
**Overall Event Rate**					
Total Events (N)	818	164	654	38	780
Total Person-Years	38,074	9,139	28,935	2,734	35,340
Incidence Rate per 100 person-years	2.15	1.79	2.26	1.39	2.21

In multivariable Cox proportional hazards models adjusting for the independent variables listed above in Methods, being adherent to either regimen (pooled) was associated with significantly lower risk of CV event (HR = 0.77, p = 0.003). In a separate model that did not adjust for adherence status, CV events were lower for SPAA than for CCB+statin patients (HR = 0.68, p = 0.02). A combined model compared 4 cohorts based on the combination of treatment and adherence status. Using non-adherent CCB + statin patients as the reference group, the risk of CV events was significantly lower among adherent CCB + statin patients (HR = 0.79, p = 0.01) and adherent SPAA patients (HR = 0.61, p = 0.03); the risk was similar for non-adherent SPAA patients (HR = 0.69, p = 0.14)..

## Discussion

As with prior analyses, CCB or statin patients who start on SPAA are more likely to be adherent to antihypertensive and statin therapy in the first six months than are patients who add a CCB to statin or a statin to CCB as 2 separate pills [10,11]. As an extension of increased adherence due to single pill advantages, this study found that greater adherence to hypertension and dyslipidemia therapy appears to have translated into a lower risk of CV events over time compared to non-adherent patients.

Slightly over 56% of the 1537 SPAA patients had at least 80% PDC adherence in the six months following initiation of therapy, compared with 21% of the 17,910 patients prescribed both a CCB and a statin. These adherence rates are consistent with other studies of single and dual-pill treatment of naïve patients with antihypertensive or statin therapy. In a study by Jackson et al., [31] the effect of additional pills was evaluated as to its impact on patient adherence to medication, specifically measured via the medication possession ratio (MPR). Findings from this study suggest that an inverse relationship exists between additional medication tablets (pills) and patient MPR, as measured among patients receiving antihypertensive therapy in a managed care setting. MPR values were reduced from 75.4% among patients with a 2-tablet amlodipine regimen to 60.5% among patients with a 3-tablet amlodipine regimen. In another study with similar adherence findings to this study, Gerbino et al. [32] also showed a positive relationship between utilization of the fixed dose regimen and patient adherence, with MPR-based adherence measured at nearly 20% less among patients with ACE inhibitors plus CCB versus patients with a fixed dose amlodipine-benazepril.

This study demonstrates that patients' risk of cardiovascular events was significantly decreased among adherent patients compared with non-adherent patients. Unadjusted results found that the 12-month cardiovascular event incidence rate was only 1.88 per 100 person-years for adherent patients compared with 2.47 per 100 person-years in non-adherent patients. Adherence to either of the regimens included in the study was associated with a significantly lower risk of CV event (HR = 0.77, p = 0.003) after adjusting for potentially confounding baseline characteristics in multivariable Cox proportional hazards models.

This association between adherence and cardiovascular risks is in agreement with previous studies, including the 2007 study by Munger et al. [33] In this review, medication nonadherence was found to be responsible for several adverse health and economic outcomes, including an increased risk of death among patients with a prior myocardial infarction, an estimated annual cost of $396 to $792 million, and 33% to 66% of medication-related hospital admissions. Sever et al. [34,35] found that three years of atorvastatin therapy produced a 79% reduction in coronary heart disease related events, from 22.8 events per 1,000 patient years to 4.8 events per 1,000 patient years. That study also found benefits of amlodipine and atorvastatin in reducing nonfatal MI by 46% [hazard ratio 0.54, confidence interval (CI) 0.40-0.72, P < 0.0001], stroke by 37% [hazard ratio 0.63, CI 0.46-0.87, P = 0.004] and total cardiovascular events and procedures by 27% [hazard ratio 0.73, CI 0.63-0.86, P < 0.0001].

This study has several limitations worth noting. Since PDC calculations are based on the assumption that patients take all medications for which they have prescriptions filled, these measures may overestimate adherence. Additionally, these adherence calculations fail to account for the possibility that patients received medications from sources other than the pharmacies included in the database used in this study.

Because of the way adherence was calculated in this analysis (patients had to remain on both CCB and statin to be considered adherent), our adherence rates may appear low relative to what has been reported in the literature. However, given that the patients in this analysis were prescribed both drugs, we believe patients should be considered nonadherent for the purposes of this study if they discontinue either CCB or statin.

Adherence was measured in a time period separate and distinct from the period during which CV events were identified and recorded. Due to this fact, it is possible that CV events occurred in the 6-month adherence measurement period and were not accounted for in our analysis, or that patient adherence measured prior to CV event monitoring is not representative of refill behavior had adherence and events been measured concurrently. To the extent that patients' adherence to their index medications differed between the 6-month period immediately following initiation of therapy and the minimum 12-month subsequent period, the estimates for adherence may vary from the time-specific values.

Cardiovascular events were identified by healthcare claims containing specific ICD-9 diagnosis codes. Due to the potential for unreported, misreported or miscoded cardiovascular events, the estimates for CV event incidence may overestimate or underestimate the actual number of clinical events. Since this limitation is similar for both cohorts, we do not expect it to bias the analysis for or against a cohort.

Two final limitations exist relative to analysis using a retrospective cohort design and adjudicated healthcare claims. Findings in this study are representative only of the U.S. commercially insured population of patients, not the overall population of treated patients who may have other forms of healthcare coverage (Medicaid, Medicare, etc.) not captured through this study methodology. Additionally, factors associated with both patient adherence and the incidence of cardiovascular events are limited within this study to those elements available through health plan enrollment files and insurance claims. Unmeasured and unknown confounding factors related to both baseline characteristics and clinical outcomes may exist, and their effect on these results cannot be accurately quantified.

## Conclusions

Patients receiving SPAA rather than a two-pill CCB + statin regimen are more likely to be adherent. In turn, adherence to CCB and statin medications was associated with lower risk of CV events in primary prevention patients.

## Competing interests

This study was sponsored by Pfizer Inc, New York, NY. Dr. R.H. Chapman and Mr. Jason Yeaw of IMS Health were paid consultants to Pfizer Inc in connection with the development of the manuscript. Dr. C.S. Roberts is an employee of Pfizer Inc. Editorial support provided by Katharine Coyle at IMS Health was funded by Pfizer Inc.

## Authors' contributions

RHC participated in the study design and analysis, and helped to draft the manuscript. JY participated in the design of the study, performed the statistical analysis, and helped to draft the manuscript. CSR conceived of the study, participated in its design, and helped to draft the manuscript. All authors read and approved the final manuscript.

## Pre-publication history

The pre-publication history for this paper can be accessed here:

http://www.biomedcentral.com/1471-2261/10/29/prepub
